# Distinct microbiota composition and dendritic cell activation in the appendix microenvironment of ulcerative colitis patients

**DOI:** 10.1080/19490976.2025.2545416

**Published:** 2025-08-19

**Authors:** Melania Scarpa, Ignazio Castagliuolo, Ilaria Patuzzi, Andromachi Kotsafti, Astghik Stepanyan, Claudia Armellin, Giovanni Tagliente, Edoardo Savarino, Fabiana Zingone, Cesare Ruffolo, Luca Saadeh, Gaya Spolverato, Imerio Angriman, Marco Scarpa

**Affiliations:** a Lab of Advanced Translational Research, Veneto Institute of Oncology (IOV-IRCCS), Padova, Italy; b Department of Molecular Medicine (DMM), University of Padova, Padova, Italy; c EuBiome S.R.L, Padova, Italy; d General Surgery Unit 3, Azienda Ospedale Università di Padova, Padova, Italy; e General Surgery Unit, Casa di Cura, Abano Terme, Italy; f General Surgery Unit, Ospedale Dell’Angelo, AULSS 3 Serenissima, Venezia, Italy; g Department of Surgical, Oncological and Gastroenterological Sciences (DISCOG), University of Padova, Padova, Italy

**Keywords:** Appendix, ulcerative colitis, microbiota, dendritic cells

## Abstract

Appendectomy has been associated with reduced risk of developing ulcerative colitis (UC) or experiencing flares after diagnosis, suggesting the appendix may play a role in UC pathogenesis. Given its function in microbial homeostasis and gut immunity, we investigated the relationship between mucosal microbiota and immune environment of the appendix in UC. Appendix tissue was collected from 85 patients undergoing surgery for UC, acute appendicitis (APA) or colon cancer (CC). Immunophenotyping of dendritic cells (DC), macrophages and T lymphocytes was performed using flow cytometry. Microbiota composition was analyzed via 16S rRNA gene amplicon sequencing. Although alpha diversity did not differ between UC and non-UC appendices, beta diversity indicated significant compositional differences. Five bacterial species (*Actinomyces hyovaginalis*, *Mogibacterium sp*. *Fusobacterium sp*. *Pseudoramibacter eubacterium*, and *Streptococcus anginosus*) were significantly reduced in the UC appendix compared to APA. However, no species were associated with UC disease activity. In contrast, UC patients showed a significantly higher frequency of activated DCs (CD1a^+^ HLAdr^+^ CD86^+^). DC activation levels correlated with daily stool frequency and T-cell activation. These findings suggest that the appendix may contribute to UC pathogenesis through immune, rather than microbial, mechanisms – supporting a role for dendritic cell-mediated T-cell priming in colonic inflammation.

## Introduction

Ulcerative colitis (UC) is a chronic inflammatory disease of the colon, confined to the mucosa, and characterized by repeated flare-ups. Pathogenesis is considered multifactorial, involving both genetic and environmental factors.^1–3^ These include living in developed countries, antibiotic use,^4,5^ intestinal infections,^6–9^ and the use of non-steroidal anti-inflammatory drugs.^10,11^ Conversely, smoking^12–14^ and appendectomy^5,15–22^ have been identified as protective factors.

Over the last 20 years, substantial evidence has emerged supporting the role of the cecal appendix in the development and course of UC. Epidemiological and case-control studies have shown an inverse relationship between prior appendectomy and the development of UC, with inflammation often observed around the cecal orifice of the appendix in UC patients, particularly those with isolated distal colitis (48–86%) without right colon involvement.^22–28^ This has led to the hypothesis that the appendix is one of the primary sites for developing UC.

However, studies aimed at evaluating the effect of appendectomy on the clinical course of UC and the risk of colectomy have yielded conflicting results. Some studies suggest that appendectomy leads to less severe disease evolution with reduction in flare-ups and decreased need for immunomodulators or colectomy.^17,18,29,30^ In contrast, other studies have found no significant benefit of appendectomy on clinical outcomes or colectomy incidence.^16,31^ Furthermore, several systematic reviews have been conducted, but they have not clarified the real effectiveness of appendectomy in influencing the evolution of UC. This is mainly due to the heterogeneity of the studies, particularly in the methods of prognosis assessments, including hospitalization, reduction in medical treatment, and the need for colectomy.^32^ Notably, the recent randomized controlled trial ACCURE, which compared appendectomy plus standard medical therapy to standard medical therapy alone for maintenance of remission in ulcerative colitis, showed a significantly lower 1-year relapse rate in the appendectomy group compared to controls.^33^

Experimental evidence from studies using T cell receptor (TCR)-α knockout mice has shown that early appendectomy suppresses inflammation in models of colitis.^34^ Further studies have demonstrated an increased proportion of CD4+CD69+ cells in the appendix of UC patients compared to controls,^23^ suggesting a role of immune cells in the appendix. Other authors have hypothesized that appendectomy alters the immune response, shifting it toward suppressor T cells by reducing the number of T helper cells, and therefore reducing the expression of interleukin-4, which is implicated in UC pathogenesis.^22,34,35^

In UC, goblet cells depletion and a defective mucin layer, which is an important element for the penetration of luminal bacteria, have been observed. It is not known whether this change is causal, but it is evident that the abnormal interaction between the immunocompetent cells of the mucosa and the intestinal flora contributes to the development of the anomalous inflammatory response in UC.^36^ Although dysbiosis is common in UC patients, its characteristics are highly variable across individuals,^37^ and specific bacteria associated with UC onset remain unidentified.^38^ Given the hypothesis that the appendix serves as a reservoir of commensal bacteria, appendectomy may alter the evolution of UC by preventing recolonization of the colon. Therefore, investigating the microbial diversity in the appendix of UC patients could provide insights into its role in triggering the immunological cascade favored by a deficient mucosal barrier.

Our working hypothesis is that the alteration of intestinal microbial flora homeostasis within the cecal appendix, and its interaction with the numerous immunocompetent cells present in this region, may play a significant role in the onset and evolution of UC. This study aims to investigate the possible relationship between mucosal microbiota in the appendix and the local inflammatory response in UC, with a focus on how these factors may contribute to disease severity.

## Methods

### Study design

This prospective observational study was conducted on a cohort of consecutive patients and was approved by the Ethical Committee of the province of Padova (CESC code: 5324/AO/22). The study was performed in accordance with the principles of the Declaration of Helsinki. This was a cross-sectional, observational study without any interventional procedures. No longitudinal data or follow-up were collected, and the study was not registered in a public clinical trial database. Prior to enrollment, each patient received detailed information about the study methods and objectives and provided written informed consent.

We compared the microbiological microenvironment and inflammatory response in the appendix of UC patients undergoing proctocolectomy, individuals who underwent surgery for acute appendicitis (APA), and patients with a normal appendix undergoing right hemicolectomy for colon cancer (CC). All UC patients were undergoing proctocolectomy, which is generally reserved for advanced, refractory, or medically uncontrolled disease. For UC patients, disease severity at the time of surgery was assessed using the Mayo score, and treatment regimens – including immunosuppressive therapies – are reported in Supplementary Table S1.

A full-thickness 3 mm slice of the appendix was collected at surgery, which was then divided into three portions: one portion was snap-frozen for microbiota analysis, another portion was sent fresh for flow cytometry, and a third portion was formalin-fixed and paraffin-embedded for histological examination.

### Sample size calculation

The primary outcome of this study was to compare the immune microenvironment in the appendix of UC, APA and CC patients. With a significance level of 0.05, power of 0.80, and a standardized effect size of 1 (as suggested by our previous studies^8–10^) the calculated sample size required for each group was 16 patients.

### Histological evaluation

For routine histological analysis, appendix samples were fixed in 4% paraformaldehyde (PFA) for 24 hours, followed by dehydration and embedding in paraffin. Sections of 5 μm thickness were cut and stained with standard hematoxylin/eosin (H&E). Inflammatory severity was quantified using the Floren scoring system.

### Flow cytometry

Appendiceal mucosa samples were washed in Hanks’ Balanced Salt Solution (HBSS) containing 10 mM dithiothreitol (DTT) and digested to obtain single-cell suspensions. Freshly isolated cells (10^5^) were stained in phosphate buffered saline (PBS) containing 2% fetal bovine serum (FBS) with appropriate combinations of FITC- and PE-conjugated antibodies. Flow cytometry was used to determine the proportion of activated (HLAdr+ and CD86+) dendritic cells (CD1a+), macrophages (CD163+), and activated T cells (CD69 expression on CD4+ and CD8+ lymphocytes). The specific antibodies used are listed in Supplementary Table S2.

### DNA extraction and 16S rRNA gene amplicon sequencing

DNA extraction from appendiceal mucosa samples was performed by using the Ultra-deep Microbiome Prep Kit (Molzym GmbH & Co. KG) according to the manufacturer’s instructions. Buffer-only extraction controls were included to monitor for reagent and environmental contamination during DNA extraction. However, no mock community controls were sequenced in parallel. As a result, while potential contaminants could be partially assessed and removed, the absence of a defined positive control limits comprehensive evaluation of sequencing accuracy and taxonomic assignment fidelity.

Sequencing was performed at BMR Genomics S.r.l. The V3–V4 regions of 16S rRNA gene were amplified using the primers Pro341F (5′-CCTACGGGNBGCASCAG-3′) and Pro805R (5′-GACTACNVGGGTATCTAATCC-3′). The primers were modified with forward and reverse overhangs for dual-index library preparation following Illumina protocol. Samples were normalized, pooled, and run on an Illumina MiSeq with a 2 × 300 bp read length.

### Bioinformatic analysis

Forward and reverse reads were preprocessed and analyzed using the Quantitative Insights into Microbial Ecology (QIIME2, version 2022.8) pipeline. Amplicon sequence variants (ASV) were identified using the DADA2 plugin, and taxonomic assignment was performed using the Greengenes database (version 13_8) with a Naive Bayes classifier trained on the V3-V4 region. To filter low-depth samples, we evaluated several thresholds for minimum read count per sample, including 15,000, and 20,000 reads. Based on rarefaction curve analysis (Supplementary Figure S1), we observed that a 20,000-read threshold offered better differentiation between groups in alpha diversity metrics, with only a minor impact on the number of samples excluded. Therefore, we retained the 20,000-read cutoff for all downstream diversity analyses to ensure comparability and robustness of results. Alpha diversity indices (Richness, Pielou, and Shannon indices) and beta diversity measures (Bray-Curtis, Jaccard, Weighted and Unweighted UniFrac) were calculated to assess microbial community diversity, with rarefaction level equal to 20,000 reads. Beta diversity measures were used for ordination analysis with the principal coordinates analysis (PCoA) technique. Alpha diversity analysis was performed via QIIME2 plugins, and graphically rendered in R (version 4.1.0), while beta diversity calculation and ordination plot production were performed in R using phyloseq (version 1.36.0) and vegan (version 2.5–7) packages. Data were normalized using the GMPR tool (version 0.1.3). Differential abundance analysis was performed at the species level using the ANCOMBC package (v 1.2.2).

### Statistical analysis

Statistical analyses were performed using Microsoft Excel and Statistica 7.1 (StatSoft, Inc.). Continuous data are expressed as median with ranges while categorical data are presented as frequencies and proportions. Non-parametric tests were used: Mann – Whitney U test for continuous variables and Fisher’s exact test for dichotomous ones. Spearman correlation tests were used to evaluate relationships between variables. Statistical significance was set at *p* < 0.05.

## Results

### Patients’ characteristics

Eighty-five patients were enrolled in this study, and samples were taken from their appendix when the subjects underwent surgery. In our cohort we enrolled 43 patients with UC, 21 with CC and 21 with APA. The most notable difference among the three groups is the disease duration, from 1 day of the APA group to over 10 years in the UC group. UC patients were receiving immunosuppressive therapy, as detailed in Supplementary Table S1. Patients’ characteristics are shown in Table 1.

**Table 1.** Patients’ characteristics.

	Acute Appendicitis		Colon Cancer		Ulcerative Colitis		
	Median	25–75 P	Median	25–75 P	Median	25–75 P	P value
Gender	M 13	F 8	M 15	F 6	M 29	F 14	P = 0.803
Age at diagnosis	40	24,25–66,75	77	66,75–78,25	53	39,75–61,00	*p* = 0.0001
Disease duration (days)	1	1,00–2,25	25,5	9,50–58,50	3940	843,0–7521,0	*p* < 0.0001
BMI	20,85	19,60–23,65	23,75	22,50–27,60	22,6	19,90–25,10	*p* = 0.227
Score AAS	9,5	9,00–14,00	5	2,75–5,00	3	2,00–6,50	*p* = 00012
Score AIR	5,5	5,00–6,00	2	1,25–2,00	2	0,00–4,25	*p* = 0.014
Daily stools	NA		NA		10,0	6,50–20,00	
Partial Mayo Score	NA		NA		4	3–5	

### Microbiota in the appendix of patients with APA, CC and UC

The analysis of the microbiota adherent to the appendix mucosa was conducted on a subgroup of 62 patients, including 30 with ulcerative colitis (UC), 17 with APA, and 15 with CC. A total of 4,086,298 raw reads were generated from sequencing the 62 samples, with a median of 61,038 reads per sample. After preprocessing and constructing the amplicon sequence variant (ASV) table, 1,180,135 reads were retained for further analysis, with a median frequency of 22,778 reads per sample. Twenty-five samples were excluded due to insufficient reads, resulting in a final set of 37 samples suitable for microbiota analysis (20 UC, 10 APA, and 7 CC).

**Figure 1.**
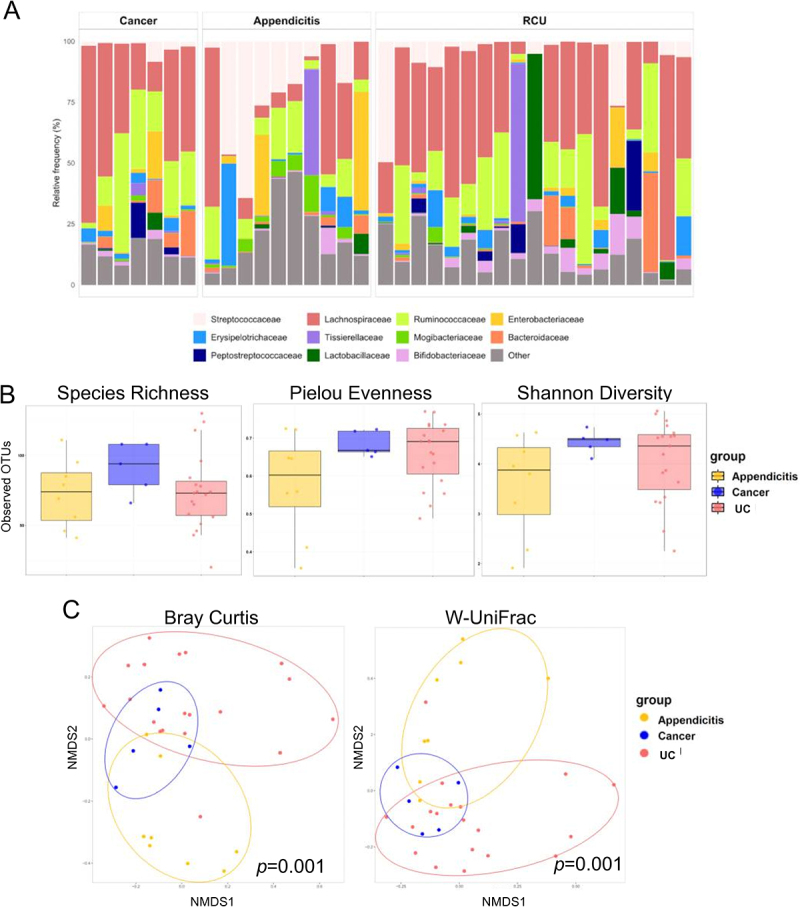
Microbiota in the appendix of patients with APA, CC and UC. (A) Bar plot showing family distribution in the appendix of APA, CC and UC patients. The top seven most abundant families per group are shown; remaining families are grouped as “other.” (B) Box plots of α-diversity between APA, CC and UC patients according to richness, Pielou eveness and Shannon Index. (C) PCoA plots of Bray-Curtis and Weighted UniFrac distance metrics according to APA, CC and UC patients.

Taxonomic profiling of the appendix mucosa samples revealed that *Firmicutes* was the most abundant bacterial phylum in UC patients, accounting for an average of 72.6%, followed by *Proteobacteria* at 16% (Figure 1(A)). After applying a rarefaction threshold of 20,000 reads, five samples were excluded from α-diversity evaluation. No significant differences in species richness, Pielou evenness, or Shannon diversity indices were observed among the three patient groups (Figure 1(B)). However, PERMANOVA tests on Bray-Curtis and Weighted UniFrac β-diversity revealed significant differences in the microbial communities of the appendix across the three groups (Figure 1(C)).

**Figure 2.**
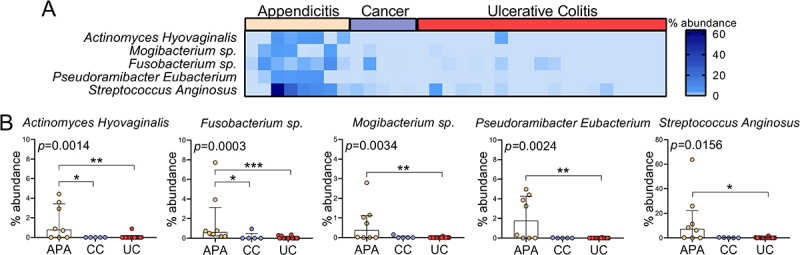
Comparison of microbial profiles in the appendix of patients with APA, CC and UC. (A) Heatmap of five differentially abundant species in the appendix of APA, CC and UC patients. (B) Relative abundance of five differentially abundant species in the appendix of APA, CC and UC patients.

Differential abundance analysis identified five bacterial species—*Actinomyces hyovaginalis, Mogibacterium sp., Fusobacterium sp., Pseudoramibacter eubacterium*, and *Streptococcus anginosus*—as differentially abundant across the three groups (Figure 2(A)). All five species were significantly less abundant in the appendix of UC patients compared to those with APA (Figure 2(B)). Notably, *Fusobacterium, Pseudoramibacter, and Mogibacterium* levels predicted UC diagnosis in models adjusted for age at diagnosis (*p* = 0.028, *p* = 0.016, and *p* = 0.042, respectively). However, age at diagnosis did not independently predict UC when tested as a covariate alongside microbial markers (data not shown). Additionally, *Fusobacterium, Pseudoramibacter*, and *Mogibacterium* were found to be differentially abundant at the genus level (data not shown). Furthermore, *Fusobacterium* abundance was positively correlated with both patients’ body mass index (BMI) (rho = 0.56. *p* = 0.024) and appendicitis severity score (rho = 0.71, *p* = 0.012). No significant associations were observed between the abundance of any microbial species and UC disease activity.

*p<0.05; **p<0.01; ***p<0.001.

### Immune microenvironment in the appendix of patients with APA, CC and UC

**Figure 3.**
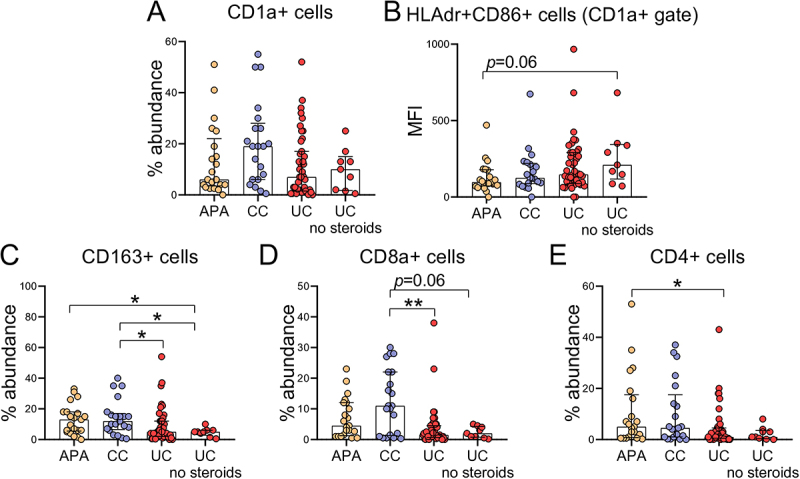
Immune microenvironment in the appendix of patients with APA, CC and UC. Flow cytometry analysis of (A) CD1a+ (B) CD1a+HLAdr+CD86+ (C) CD163+ (D) CD8a+ (E) CD4+ cells in the appendix of patients with APA, CC and UC.

The abundance of dendritic cells (DCs, CD1a+), M2 macrophages (CD163+), and CD4+ and CD8+ T lymphocytes were quantified in the appendiceal mucosa of all collected samples. The proportion of DCs was generally lower in UC patients (*p* = 0.07, Figure 3(A)); however, the mean fluorescence intensity of HLAdr+CD86+ DCs (activated DCs) was higher in the UC appendix compared to the APA group, particularly among patients who were not receiving steroid treatment (*p* = 0.06, Figure 3(B)). The abundance of macrophages (CD163+) and cytotoxic T lymphocytes (CD8+) was significantly lower in UC patients compared to those with CC (Figure 3(C,D)). This difference remained evident when the analysis was restricted to patients not receiving steroid therapy (*p* = 0.02, Figure 3(C), and *p* = 0.06, Figure 3(D)). Additionally, T helper cells were significantly reduced in the UC patients’ appendix (Figure 3(E)). Notably, M2 macrophages (CD163^+^), T helper lymphocytes (CD4^+^), and cytotoxic T lymphocytes (CD8a^+^) all predicted UC diagnosis in models adjusted for age at diagnosis (*p* = 0.055, *p* = 0.013, and *p* = 0.001, respectively). However, age at diagnosis did not independently reach statistical significance as a covariate in these models (data not shown).

*p<0.05; **p<0.01

### Activated dendritic cells in the appendix of patients with UC

**Figure 4.**
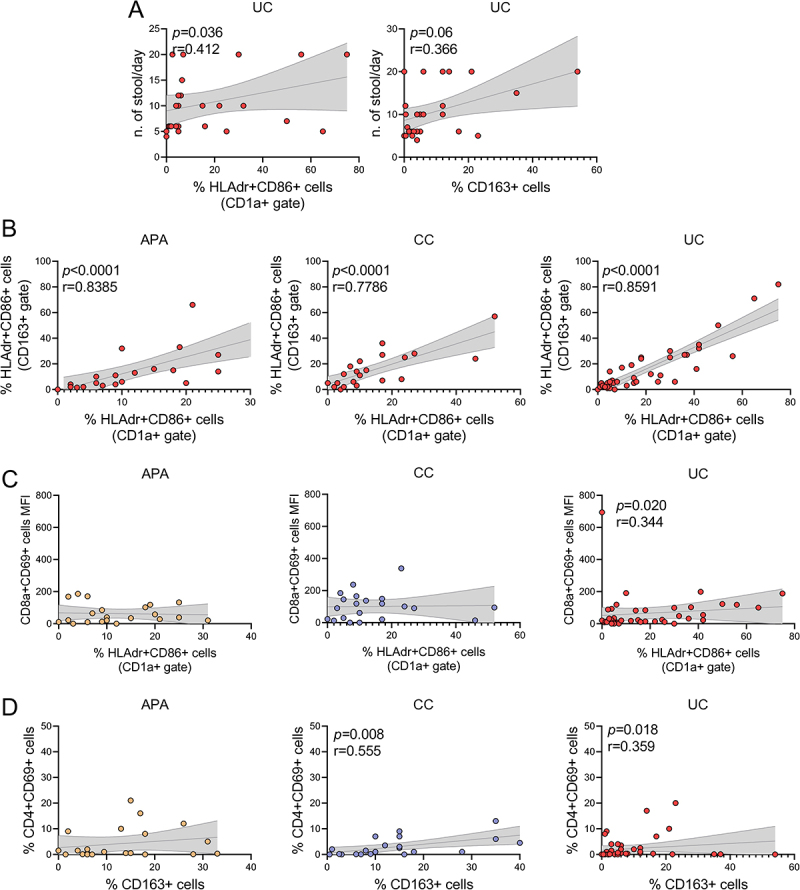
Activated dendritic cells in the appendix of patients with UC. Direct correlation between activated DC and M2 macrophages and (A) Number of daily stools. (B) Correlation between activated DC and activated M2 macrophages in the appendix of patients with APA, CC and UC. (C) Correlation between activated DC and activated CD8a+ lymphocytes mean fluorescence intensity in the appendix of patients with APA, CC and UC. (D) Correlation between M2 macrophages and activated CD4+ lymphocytes in the appendix of patients with APA, CC and UC.

In UC patients, the rates of activated DCs and M2 macrophages were directly correlated with the number of daily stools (Figure 4(A)). However, no correlation was found with disease duration. Moreover, the rate of activated DCs resulted directly correlated with the rate of activated M2 macrophages in all three groups of patients (Figure 4(B)). Notably, in UC patients – though not in those with APA or CC – the rate of activated DCs was directly correlated with the mean fluorescence intensity of activated cytotoxic T lymphocytes (Figure 4(C)). Additionally, the rate of M2 macrophages was directly correlated with the rate of activated T helper lymphocytes in both UC and CC patients, but not in APA patients (Figure 4(D)). The interaction between immunosuppressive therapy and the immune microenvironment within the appendiceal mucosa is detailed in Supplementary Table S3.

## Discussion

Recent evidence suggests that appendectomy may reduce the risk of developing UC or even the risk of flare after UC is diagnosed, suggesting a potential involvement of the appendix in UC pathogenesis. Several observational studies have shown that appendectomy is associated with a less severe disease evolution with reduction in flare-ups, decreased use of immunomodulators, and lower rates of colectomy^17,18,29,30^. In fact, the ACCURE trail confirmed the protective role of appendectomy against UC relapse, reporting a relative risk 0.65 [95% CI 0.47–0.89]^33^. Interestingly, several studies support the role of the appendix in the maintenance of the intestinal microbiome and gut immunity^39^, with patients who have had their appendix removed showing reduced gut microbiota richness and diversity^40^. Thus, our first hypothesis was that the appendix in UC patients may function as a reservoir for an altered microbiota that could promote dysbiosis that drive inflammation in the colon.

Our analysis of the microbiota adherent to the appendix mucosa revealed that *Firmicutes* was the most abundant bacterial phylum in the UC appendix, followed by *Proteobacteria*. Alpha diversity analysis showed no significant differences in species richness, Shannon, and Pielou indices between UC and non-UC appendices. Similarly, Oh et al^41^ found that the relative abundances of the major phyla (*Firmicutes, Proteobacteria, Bacteroidetes*, and *Actinobacteria*) in appendiceal tissues were similar in patients with APA and those with non-inflamed appendices. At first glance, these results suggest that the microbiota plays a minimal role in UC inflammation. In our series, we observed significant differences in the bacterial communities of the UC and non-UC appendix. Specifically, we identified five species that were differentially abundant, all of which were significantly reduced in UC appendices compared to non-UC appendices: *Actinomyces hyovaginalis, Mogibacterium sp., Fusobacterium sp., Pseudoramibacter eubacterium*, and *Streptococcus anginosus*. Notably, *Fusobacterium sp*., *Pseudoramibacter eubacterium* and *Mogibacterium sp*. were also differentially abundant at the genus level. These findings are consistent with those of Swidsinski et al^42^, who demonstrated that local infection with *Fusobacterium nucleatum/necrophorum* is responsible for the majority of cases of APA. The prevalence of *Fusobacterium* is also linked to the severity of appendix inflammation^43^, and this genus has been shown to persist even after several weeks of broad-spectrum antibiotics treatment prior to surgery^44^. Thus, our data support the role of a distinct microbiota profile in APA while the microbiota of the UC-affected appendix appears to be characterized by the absence of certain species or genera. Moreover, in an experimental model of DSS-induced colitis, microbiota analysis revealed a comparable fecal microbiota profile one week post-surgery between the appendectomy and control groups^45^. These observations suggest that the potential effect of appendiceal microbiota in UC may be limited. In conclusion, although the small and unbalanced sample size reduces the statistical power of our microbiota analyses, our data do not provide clear evidence for a major role of the appendiceal microbiota in UC pathogenesis. Moreover, taxonomic assignments at the species level based on 16S rRNA gene sequences can sometimes be inaccurate, particularly for taxa with limited representation in reference databases. Some species identified – such as *Actinomyces hyovaginalis*—may therefore reflect misannotation rather than true presence in the human appendiceal mucosa. Another limitation of this study is the absence of mock community controls in the sequencing workflow. Although buffer-only extraction controls were used to identify and exclude likely contaminants, the lack of a defined synthetic community prevents a more systematic assessment of sequencing error, taxonomic resolution, and inter-batch variability. Finally, we acknowledge that 16S rRNA gene amplicon sequencing has limited taxonomic resolution, particularly at the species or strain level. While our approach is validated and widely used for profiling mucosa-associated microbiota, metagenomic sequencing would have enabled more precise taxonomic and functional characterization. This represents an important area for future investigation. Thus, while our data do not support a major role of the appendix-associated microbiota in UC pathogenesis, these findings remain exploratory.

Given this, we next focused on the hypothesis that underlying immune dysregulation in the appendix may contribute more directly to UC onset. In our series, in the appendiceal mucosa, macrophages cell rate (CD163+), T helper lymphocytes (CD4+), T cytotoxic lymphocytes (CD8+) and activated cytotoxic lymphocytes (CD8+CD69+) were all significantly lower in UC patients compared to appendicitis and CC patients. Thus, we can reasonably exclude these cell populations as the potential drivers of colonic inflammation. This also appears to be true for M2 macrophages, which, despite their cell number being directly correlated with the rate of activated T helper cells, were found to be lower in UC compared to APA and CC.

Interestingly, although total DCs (CD1a+) tended to be lower in UC patients, activated DCs (CD1a+HLAdr+CD86+) tended to be higher in the UC group compared to APA patients and this was even more significant in UC patients who did not have any steroids therapy. Waraich et al^46^ also observed that in UC, but not in APA, there was an extension of a network of dendritic cells into the crypt mucosa, which were closely aligned with the epithelium, that showed intense upregulation of HLA class II. The presence of this subpopulation, in close contact with the epithelium showing an altered expression of HLA class II antigens, suggests that this component of the immune response is targeting this area in UC.

In our series, in UC patients, activated DCs (CD1a+HLAdr+CD86+) rate directly correlated with disease activity, as assessed by the number of daily stools. Moreover, we found that activated DCs correlated with activated T cytotoxic lymphocytes (CD8+CD69+) and activated macrophages (CD163+HLAdr+CD86+), suggesting a potential role in T-cell priming. In the study by Collard et al^45^, at the DSS treatment endpoint (DSS-only protocol), CD3+ and CD8+ T-cell densities in the colonic lamina propria were significantly reduced following appendectomy, suggesting that immune response coordination in the colon may occur within the appendix. These findings support the idea of colonic T-cell priming in the appendix, although other mechanisms may also be involved^47,48^. Our data suggest that this role might be attributed to activated DCs. However, we recognize that these findings are correlative and do not establish a direct causal relationship between activated dendritic cells and T-cell priming in UC pathogenesis. Functional validation – such as cytokine profiling or T-cell co-culture assays – would be required to confirm the capacity of these cells to drive immune activation. Given the observational and exploratory nature of this study, along with the inherent limitations of using surgical tissue samples, such mechanistic investigations were beyond the scope of the current work. Therefore, these results should be considered hypothesis-generating and warrant further in-depth functional studies.The co-stimulatory molecule CD86, associated with HLA class II, plays a pivotal role in T-cell priming by regulating the activation of T lymphocytes via its interaction with CD28. In our previous study, we demonstrated that CD86 expression correlates with the histological grade of disease and with serum CRP levels, with upregulation occurring early in the disease^49^. Recent genome-wide association studies have identified CD28 as a susceptibility locus for several autoimmune diseases, UC and primary biliary cholangitis^50^. Interestingly, the immune checkpoint inhibitor Ipilimumab (which targets cytotoxic T-lymphocyte-associated antigen-4, CTLA-4, the inhibitory receptor of CD86^51^ has been associated to immunotherapy complications such as Ipilimumab-associated colitis (Ipi-AC), an immune-mediated colitis that mimics inflammatory bowel disease^52^. Although Ipi-AC is a distinct pathologic entity with some clinical and histopathological peculiarities, it shares several features with UC, particularly the same mucosal infiltration of CD4+ and CD8+ T-cells^52^. These findings suggest that dendritic cells expressing CD86 in the appendix may play a critical role in initiating T-cell activation in the colon in UC patients.

A strong limitation of this study is the variation in disease duration among groups, ranging from 1 day in the APA group to over 10 years in the UC group. This could influence the inflammatory patterns observed, as disease progression may alter the immune response over time. Additionally, differences in age and sex distribution between cohorts represent potential confounders, particularly for immunophenotypic and microbiota analyses, and should be taken into account when interpreting the results. In our previous study on newly diagnosed Crohn’s disease, we observed high levels of IL-15 and IL-23 in healthy mucosa, along with neutrophil infiltration, which are likely the starters of acute inflammation^53^. Moreover, evaluating the potential impact of activated DCs on the UC activity index over a longer follow-up period would have been of considerable interest. However, as appendix samples were collected at the time of proctocolectomy, the clinical course of UC effectively concluded for each patient at that point, precluding longitudinal assessment. In conclusion, our study suggests that appendiceal microbiota may not play a major role in UC pathogenesis. However, we found that the immune profile in the appendiceal mucosa of UC patients, particularly the activation of DC expressing CD86, may contribute to T-cell priming in the colon and thus to the pathogenesis of UC.

## Supplementary Material

Supplementary Figure 1 rev1.tif

Supplementary Table S1 rev1.docx

Supplementary Table S3 rev1.docx

Supplementary Table S2 rev1.docx

## Data Availability

The sequencing data generated and analyzed in this study have been deposited in the NCBI Sequence Read Archive (SRA) database under accession number PRJNA1256849. The data will be made publicly available upon publication.
