# Supplementary figures and images for: Distinct microbiota composition and dendritic cell activation in the appendix microenvironment of ulcerative colitis patients
Source: Gut Microbes. 2025 Aug 19;17(1):2545416. doi: 10.1080/19490976.2025.2545416 (PMC12366829; doi:10.1080/19490976.2025.2545416)

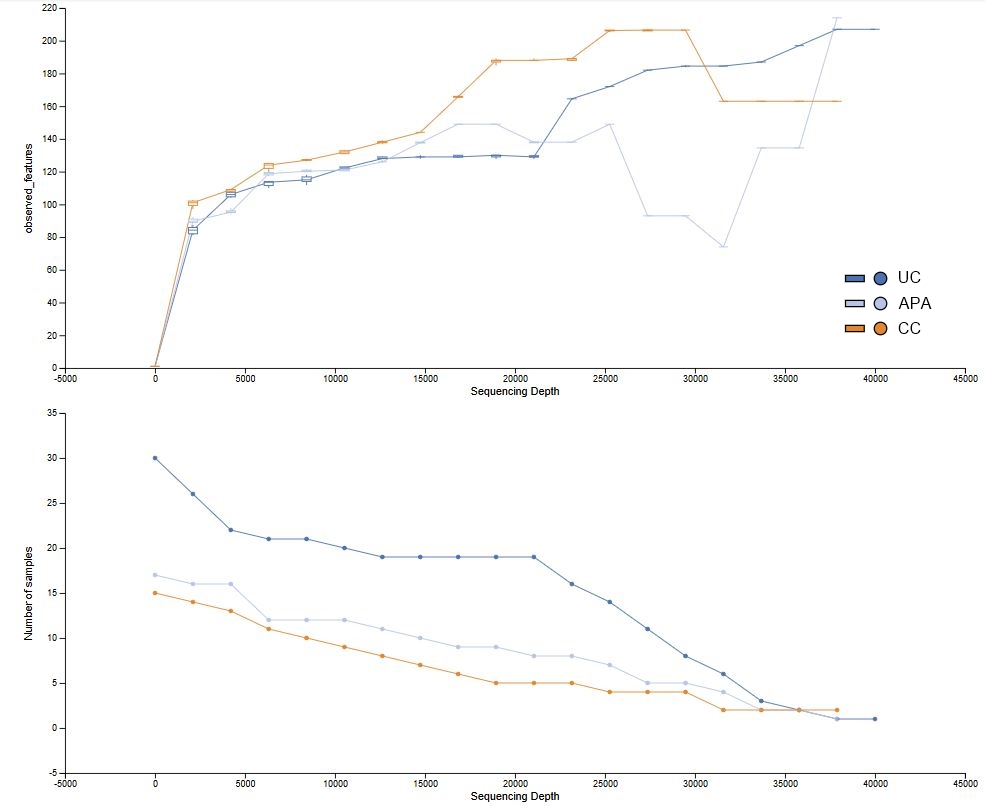

Supplement: Supplementary Figure 1 rev1.tif [file KGMI_A_2545416_SM8536.tif]
