# Supplementary material for: Distinct microbiota composition and dendritic cell activation in the appendix microenvironment of ulcerative colitis patients
Source: Gut Microbes. 2025 Aug 19;17(1):2545416. doi: 10.1080/19490976.2025.2545416 (PMC12366829; doi:10.1080/19490976.2025.2545416)
Supplement: Supplementary Table S1 rev1.docx [file KGMI_A_2545416_SM8535.docx]

**Supplemenatry Table S1**. Patients’ therapy.

| **Patients code** | **Azathioprine** | **Mesalamine** | **Vedolizumab** | **Adalimumab** | **Golimumab** | **Infliximab** | **Mercaptopurine** | **Steroids** | **Cyclosporin** |
| --- | --- | --- | --- | --- | --- | --- | --- | --- | --- |
| UC 1 | 0 | 1 | 0 | 0 | 0 | 1 | 0 | 1 | 1 |
| UC 2 | 0 | 1 | 0 | 0 | 0 | 0 | 0 | 0 | 0 |
| UC 3 | 0 | 0 | 0 | 0 | 0 | 1 | 0 | 1 | 0 |
| UC 4 | 0 | 1 | 1 | 0 | 0 | 0 | 0 | 1 | 0 |
| UC 5 | 0 | 0 | 0 | 0 | 0 | 0 | 0 | 0 | 0 |
| UC 6 | 1 | 1 | 0 | 1 | 1 | 1 | 0 | 1 | 1 |
| UC 7 | 0 | 0 | 0 | 0 | 0 | 0 | 0 | 0 | 0 |
| UC 8 | 1 | 1 | 0 | 0 | 1 | 1 | 1 | 1 | 0 |
| UC 9 | 0 | 0 | 1 | 0 | 0 | 1 | 0 | 1 | 0 |
| UC 10 | 0 | 1 | 1 | 0 | 0 | 0 | 0 | 1 | 0 |
| UC 12 | 0 | 0 | 0 | 1 | 0 | 0 | 0 | 0 | 0 |
| UC 13 | 0 | 1 | 0 | 0 | 0 | 0 | 0 | 1 | 0 |
| UC 14 | 0 | 0 | 1 | 0 | 0 | 1 | 0 | 1 | 0 |
| UC 15 | 0 | 1 | 0 | 0 | 0 | 0 | 0 | 1 | 0 |
| UC 16 | 0 | 1 | 1 | 0 | 0 | 1 | 0 | 1 | 0 |
| UC 17 | 0 | 1 | 1 | 0 | 0 | 0 | 0 | 1 | 0 |
| UC 18 | 0 | 1 | 0 | 0 | 0 | 0 | 0 | 0 | 0 |
| UC 19 | 0 | 1 | 0 | 0 | 0 | 1 | 0 | 1 | 0 |
| UC 21 | 1 | 0 | 1 | 1 | 0 | 1 | 0 | 1 | 0 |
| UC 22 | 0 | 1 | 0 | 0 | 0 | 1 | 0 | 1 | 0 |
| UC 23 | 0 | 1 | 0 | 0 | 0 | 0 | 0 | 1 | 0 |
| UC 24 | 0 | 1 | 0 | 0 | 0 | 0 | 0 | 0 | 0 |
| UC 25 | 1 | 1 | 1 | 0 | 0 | 1 | 0 | 1 | 0 |
| UC 26 | 1 | 1 | 1 | 1 | 0 | 1 | 0 | 1 | 0 |
| UC 27 | 1 | 1 | 1 | 0 | 0 | 1 | 0 | 1 | 0 |
| UC 28 | 1 | 0 | 1 | 1 | 0 | 1 | 0 | 1 | 0 |
| UC 29 | 1 | 0 | 1 | 1 | 0 | 1 | 0 | 1 | 0 |
| UC 30 | 0 | 0 | 1 | 0 | 0 | 1 | 0 | 1 | 0 |
| UC 31 | 1 | 1 | 0 | 1 | 0 | 1 | 0 | 1 | 0 |
| UC 32 | 0 | 1 | 1 | 1 | 0 | 1 | 0 | 1 | 0 |
| UC 33 | 1 | 1 | 1 | 1 | 0 | 1 | 0 | 1 | 0 |
| UC 34 | 1 | 1 | 1 | 0 | 1 | 1 | 0 | 1 | 0 |
| UC 35 | 1 | 1 | 1 | 1 | 0 | 1 | 0 | 1 | 0 |
| UC 36 | 1 | 1 | 1 | 1 | 0 | 1 | 0 | 0 | 0 |
| UC 37 | 0 | 1 | 1 | 0 | 0 | 1 | 0 | 1 | 0 |
| UC 38 | 1 | 1 | 1 | 0 | 0 | 0 | 0 | 0 | 0 |
| UC 39 | 0 | 1 | 0 | 0 | 0 | 0 | 0 | 1 | 0 |
| UC 40 | 0 | 1 | 0 | 0 | 0 | 1 | 0 | 1 | 0 |
| UC 41 | 1 | 1 | 1 | 1 | 0 | 1 | 0 | 1 | 0 |
| UC 42 | 0 | 0 | 0 | 0 | 0 | 1 | 0 | 0 | 0 |
| UC 43 | 0 | 1 | 1 | 0 | 0 | 1 | 0 | 1 | 0 |
| UC 44 | 1 | 1 | 1 | 0 | 0 | 1 | 0 | 1 | 0 |
| UC 45 | 1 | 1 | 0 | 0 | 0 | 0 | 0 | 1 | 0 |
