# Supplementary material for: Distinct microbiota composition and dendritic cell activation in the appendix microenvironment of ulcerative colitis patients
Source: Gut Microbes. 2025 Aug 19;17(1):2545416. doi: 10.1080/19490976.2025.2545416 (PMC12366829; doi:10.1080/19490976.2025.2545416)
Supplement: Supplementary Table S3 rev1.docx [file KGMI_A_2545416_SM8533.docx]

**Supplementary Table S3.** Interaction between therapy and immune activation parameters.

| **Immune markers** | **statistics** | **Azathioprine** | **Mesalazine** | **Vedolizumab** | **Adalimumab** | **Golimumab** | **Infliximab** | **Mercaptopurine** | **Steroids** | **Cyclosporine** |
| --- | --- | --- | --- | --- | --- | --- | --- | --- | --- | --- |
| CD1a+ % | Spearman's rho | -0.269 | -0.097 | 0.047 | -0.143 | -0.192 | -0.318 | -0.119 | -0.032 | -0.098 |
|  | *p-value* | *0.081* | *0.536* | *0.764* | *0.362* | *0.217* | *0.038* | *0.449* | *0.837* | *0.531* |
| CD1a+HLAdr+Cd86+ % | Spearman's rho | -0.279 | -0.262 | -0.141 | -0.083 | -0.012 | 0.143 | -0.264 | 0.117 | 0.338 |
|  | *p-value* | *0.081* | *0.102* | *0.386* | *0.612* | *0.940* | *0.378* | *0.100* | *0.473* | *0.033* |
| CD1a+HLAdr+Cd86+ MFI | Spearman's rho | 0.142 | 0.071 | 0.293 | 0.232 | -0.291 | -0.106 | -0.255 | -0.191 | -0.098 |
|  | *p-value* | *0.364* | *0.652* | *0.056* | *0.135* | *0.059* | *0.498* | *0.099* | *0.219* | *0.532* |
| CD163+ % | Spearman's rho | -0.133 | -0.159 | 0.160 | -0.212 | -0.125 | -0.008 | -0.062 | 0.074 | -0.049 |
|  | *p-value* | *0.396* | *0.307* | *0.305* | *0.173* | *0.423* | *0.960* | *0.691* | *0.637* | *0.755* |
| CD163+HLAdr+CD86+ % | Spearman's rho | -0.202 | -0.345 | -0.113 | -0.134 | 0.015 | 0.079 | -0.249 | 0.076 | 0.286 |
|  | *p-value* | *0.194* | *0.024* | *0.470* | *0.391* | *0.925* | *0.615* | *0.107* | *0.627* | *0.063* |
| CD163+HLAdr+CD86+ MFI | Spearman's rho | -0.199 | -0.137 | 0.184 | -0.046 | -0.250 | -0.124 | -0.249 | -0.150 | -0.267 |
|  | *p-value* | *0.200* | *0.379* | *0.237* | *0.770* | *0.106* | *0.429* | *0.108* | *0.338* | *0.083* |
| CD4+ % | Spearman's rho | -0.239 | 0.009 | -0.252 | -0.343 | -0.103 | -0.217 | -0.058 | 0.050 | 0.060 |
|  | *p-value* | *0.122* | *0.955* | *0.103* | *0.024* | *0.513* | *0.162* | *0.713* | *0.751* | *0.704* |
| CD4+CD69+ % | Spearman's rho | -0.317 | 0.045 | -0.300 | -0.304 | -0.109 | -0.258 | -0.130 | -0.013 | 0.025 |
|  | *p-value* | *0.038* | *0.775* | *0.050* | *0.048* | *0.485* | *0.095* | *0.405* | *0.936* | *0.876* |
| CD4+Cd69+ MFI | Spearman's rho | -0.282 | -0.120 | -0.282 | -0.220 | 0.232 | -0.276 | 0.261 | 0.025 | 0.209 |
|  | *p-value* | *0.067* | *0.442* | *0.067* | *0.157* | *0.134* | *0.074* | *0.090* | *0.872* | *0.178* |
| CD8a+ % | Spearman's rho | -0.335 | 0.002 | -0.107 | -0.403 | -0.149 | -0.277 | -0.126 | 0.035 | -0.090 |
|  | *p-value* | *0.028* | *0.989* | *0.495* | *0.007* | *0.339* | *0.072* | *0.420* | *0.823* | *0.564* |
| CD8a+Cd69+ % | Spearman's rho | -0.227 | 0.096 | -0.210 | -0.283 | -0.120 | -0.399 | -0.142 | -0.107 | -0.203 |
|  | *p-value* | *0.144* | *0.542* | *0.177* | *0.066* | *0.444* | *0.008* | *0.365* | *0.493* | *0.192* |
| CD8a+Cd69+ MFI | Spearman's rho | -0.355 | -0.157 | -0.382 | -0.257 | 0.258 | -0.313 | 0.261 | -0.044 | 0.285 |
|  | *p-value* | *0.020* | *0.315* | *0.012* | *0.096* | *0.095* | *0.041* | *0.090* | *0.780* | *0.064* |
