# Supplementary material for: Distinct microbiota composition and dendritic cell activation in the appendix microenvironment of ulcerative colitis patients
Source: Gut Microbes. 2025 Aug 19;17(1):2545416. doi: 10.1080/19490976.2025.2545416 (PMC12366829; doi:10.1080/19490976.2025.2545416)
Supplement: Supplementary Table S2 rev1.docx [file KGMI_A_2545416_SM8532.docx]

**Supplementary Table S2. Antibodies used in the study.**

| **Target** | **Conjugate** | **Clone** | **Company** |
| --- | --- | --- | --- |
| CD1a | PE | HI149 | ImmunoTools GmbH |
| CD4 | PE-Cy7 | RPA-T4 | eBioscience |
| CD8 | FITC | RPA-T8 | eBioscience |
| CD69 | PE | FN50 | eBioscience |
| CD86 | Alexa-Fluor 488 | IT2.2 | eBioscience |
| CD163 | PE | GHI/61 | eBioscience |
| HLAdr | PE-Cy7 | LN3 | eBioscience |
